# Evaluation of an Immunoturbidometric Assay for Measurement of Total Adiponectin Concentration in Equine Serum Samples

**DOI:** 10.1111/vcp.70118

**Published:** 2026-07-22

**Authors:** Rose D. Nolen‐Walston, Caitrin R. Lowndes, Jeaneen C. Kulp, Daniela Luethy, Darko Stefanovski, Andrew W. Van Eps

**Affiliations:** ^1^ Department of Clinical Studies—New Bolton Center, School of Veterinary Medicine University of Pennsylvania Philadelphia Pennsylvania USA

**Keywords:** endocrinopathy, equine metabolic syndrome, horse, insulin dysregulation, laminitis

## Abstract

**Background:**

Total adiponectin (TA) is a marker of metabolic health in equids, with values below a critical decision point of < 7.9 μg/mL conferring increased risk of laminitis.

**Objectives:**

To evaluate an IT assay to measure serum [TA] in equids and to assess its correlation with [high molecular weight adiponectin] (HMWA) measured by ELISA.

**Methods:**

Total serum adiponectin concentrations from 207 adult horses were measured using both an IT assay for TA and HMWA using a previously validated ELISA assay. Data were analyzed using Spearman correlation and Passing‐Bablok regression. Intra‐ and inter‐assay precision, linearity, lower limit of quantification (LLoQ), and freeze–thaw stability were assessed for the [TA] assay.

**Results:**

Mean (range) intra‐assay coefficient of variation (CV%) was 2.3% (1.8%–2.8%); inter‐assay, across‐day CV% was 3.3% (3.25%–3.4%), and across reagent lot CV was 11.7% (9.0%–17.9%). Mean recovery upon dilution was 133.7% (122.6%–140.6%), and LLoQ 1.3 μg/mL. The median CV for 5 freeze–thaw cycles was 3.0% (range 1.0%–3.0%) with a median change in concentration of −11% (range −[9.9–17.1]%). A strong correlation between [TA] and [HMWA] is observed (*ρ* = 0.90 [95% confidence interval 0.86–0.92], *p* < 0.001), although in 18% of samples, [HMWA] > [TA].

**Conclusions:**

The turbidimetric immunoassay is sufficiently precise and reliable for measurement of [TA] in equids, especially around the critical decision point, and LLoQ was below the lowest clinical cut‐off value. [TA] overall correlated well with [HMWA] in this population, although instances of higher [HMWA] than [TA] were noted at high [HMWA].

## 1 Introduction

Adiponectin is a peptide hormone secreted predominantly by white adipose tissue, first described in the mid‐1990s by multiple groups independently [1, 2]. This adipokine is now recognized as a key regulator of glucose and lipid metabolism, with insulin‐sensitizing, anti‐inflammatory, and anti‐atherogenic properties [1, 3, 4]. Structurally, adiponectin comprises a C‐terminal globular domain and an N‐terminal collagen‐like domain, which together allow the protein to form various multimeric complexes, including trimers, hexamers (low molecular weight), and high molecular weight (HMW) multimers [1, 5]. Among these, the HMW form is believed to be the most biologically active, particularly in modulating insulin sensitivity and inflammatory responses [3, 6].

In human and rodent studies, adiponectin has been inversely associated with obesity, insulin resistance, type 2 diabetes mellitus, cardiovascular disease, and metabolic syndrome [3, 6, 7]. Low circulating adiponectin levels, especially of the HMW isoform, are considered predictive biomarkers for these conditions and are widely used in both clinical and research settings to evaluate metabolic health [6, 8]. In equine medicine, adiponectin has been recognized for its potential as a biomarker for equine metabolic syndrome (EMS), a condition characterized by obesity, insulin dysregulation, and an increased risk of laminitis [9, 10, 11, 12, 13, 14, 15]. Similar to findings in other species, horses with EMS often exhibit hypoadiponectinemia, with The Equine Endocrinology Group suggesting a cutoff of < 7.9 μg/mL [TA] (method not specified) being supportive of a diagnosis of EMS as well as conferring increased risk of laminitis [9].

The development and validation of assays to measure these adiponectin isoforms are critical for advancing their diagnostic utility. Various assays have been used to measure adiponectin concentration in horses, including radioimmunoassay (RIA), immunoturbidometric assays (IT), and ELISA for both total and HMW adiponectin. While several human ELISA kits have been adapted for equine samples to quantify [HMWA] and [TA] [11, 16], challenges remain regarding inter‐assay variability, cross‐reactivity, and the lack of species‐specific reagents. A turbinimetric assay for TA has been validated and widely used in horses (ADPN Adiponectin immunoturbidometric assay, Randox Laboratories) [17] but is no longer commercially available. Since recommendations for the clinical use of adiponectin as a screening tool for EMS and laminitis risk are based on measurement of TA (not HMWA), there is a need to validate another TA assay for clinical use. Furthermore, multiple studies identify the need for standardized, validated tools to ensure consistent measurement across clinical and research contexts [11, 16, 17, 18, 19].

From a clinical and practical standpoint, implementation of this IT‐based platform could substantially improve the feasibility of adiponectin testing in equine practice. The assay is simple to operate, automated, high‐throughput, and inexpensive, so laboratories can provide rapid turn‐around at markedly lower costs than plate‐based ELISAs. Reduced analytical time and workflow complexity lower the barrier for routine clinical adiponectin measurement, enabling use not just as a research tool but as a screening or monitoring biomarker for clinical caseload. In particular, the ability to generate same‐day results at reasonable cost may facilitate early identification of horses with low adiponectin phenotypes who are at increased risk of endocrinopathic laminitis and allow more frequent monitoring of response to diet/exercise interventions in equine metabolic syndrome. Consequently, this platform has the potential to allow adiponectin monitoring to be added to insulin measurement as a practical clinical tool that supports proactive risk stratification and management in ambulatory and referral settings.

This study aims to assess and validate a turbidimetric human total adiponectin assay for equine plasma and serum samples; although this assay has been used in other studies, validations have not been previously published. The study aims a special focus on test performance around the broader range of 2.5–10 μg/mL adiponectin, where values > 10 μg/mL are associated with low risk of laminitis, and values < 4 μg/mL are strongly associated with increased laminitis risk [14]. A secondary aim was to correlate the results of this assay with a previously validated HMWA ELISA assay.

## 2 Materials and Methods

Study material comprised a total of 207 blood samples from adult university‐owned horses at New Bolton Center, submitted for baseline insulin measurement (not within 4 h of receiving grain but with access to hay or pasture). Blood was collected into plain vacuum tubes and allowed to clot at room temperature for 30 min, followed by centrifugation (1300 × *g*) for 10 min. Serum was harvested, transferred to 2 mL cryovials, and stored at −80°C until analysis (0–3 months).

For the experimental data, samples were analyzed using a commercial turbidimetric immunoassay, the Immunodiagnostics Adiponectin Turbidimetric Immunoassay Kit (Ontario, Canada), previously validated for the quantification of [TA] in human serum and plasma samples. In this assay, microparticles coated with an adiponectin antibody aggregate in proportion to the concentration of adiponectin and are measured by light scatter absorbance using a chemistry analyzer. Reference samples provided by the manufacturer (recombinant human origin) were used to create a standard curve, from which the concentration of patient samples can be interpolated. Specifically, 192 μL of reagent 1 (proprietary formula) is dispensed into a cuvette, mixed with a 2 μL aliquot of the serum sample, and incubated at 37°C for 5 min. A 64 μL volume of reagent 2 (ditto) is then mixed with the sample and the change in absorbance at 570 nm is read for 8 min using a custom channel on the DiaSys respons910VET (Wixom, MI) chemistry analyzer. This analyzer was installed and maintained according to the manufacturer's instructions. Daily quality control was performed using commercial 2‐level quality control materials from the kit manufacturer.

As a reference method, all samples were analyzed using the MilliporeSigma HMW Adiponectin ELISA kit, performed according to the manufacturer's instructions. This assay is a sandwich ELISA originally designed for human samples but validated for equine samples [11] and subsequently used in multiple published equine studies [10, 16, 17, 18]. In the original validation, the assay was found to have an intraassay CV of 9%–11% and interassay CV of 11%–13%, varying with concentration; spike‐recovery and linearity on dilution were 94%–109% and 72%–109% with *R* ^2^ = 0.99, respectively [11].

### 2.1 Validation of Total Adiponectin Turbidimetric Immunoassay

All 207 samples described above were analyzed using both the TA IT and HMWA ELISA assays, and had concentrations from 1.2–29.49 μg/mL and 0–37.75 μg/mL, respectively. Those samples with values appropriate for use in the validation study were employed to assess assay performance.

### 2.2 Intra‐ and Inter‐Assay Precision

Intra‐assay precision was assessed with two experiments. In the first experiment, 2 samples (5.5 and 9.0 μg/mL TA), chosen to bracket the critical decision point of 7.9 μg/mL, were assayed for a total of 20 replicates in a single session (within‐day) using the same reagent lot and operator. Additionally, in a second experiment, 10 replicates were run on each of 5 samples, also using the same reagent lot and operator, in a single session. These samples were selected to have very low (< 4 μg/mL), low (4–8 μg/mL), mid (8–10 μg/mL), high (10–12 μg/mL), and very high (> 12 μg/mL) concentrations of TA.

Inter‐assay variability was also assessed by two methods. For the first experiment, two samples were assayed once daily (across‐day) for 20 consecutive days using frozen and thawed aliquots of 2 samples with [TA] of 5.8 and 10.8 μg/mL. In a second experiment (across‐lots), 5 aliquoted, frozen and thawed serum samples with TA of 2.4–18.6 μg/mL were assayed on 4 separate occasions over a period of 10 months using reagents from 4 different lots.

### 2.3 Recovery Upon Dilution and Linearity Under Dilution

To assess recovery upon dilution and linearity under dilution, 4 banked serum samples with [TA] ranging from 4.3 to 18.6 μg/mL were aliquoted and then diluted to 66.7% (2:1), 50% (1:1), 25% (1:4), and 16.7% (1:6) concentrations with normal saline. Assays were run on undiluted aliquots and each set of serial dilutions in the same batch, with the same operator. Expected concentration was compared to the measured assay result, percentage recovery was calculated, and linearity under dilution was assessed.

### 2.4 Freeze–Thaw Stability

Three clinical serum samples with very low (< 4 μg/mL), mid (8–10 μg/mL), and very high (> 12 μg/mL) [TA] were aliquoted and frozen at −80°C. Over 5 days, the aliquots underwent 5 freeze–thaw cycles and were analyzed using the IT assay as described.

### 2.5 Lower Limit of Quantification

The lower limit of quantification (LLoQ) was defined as the lowest analyte concentration with an imprecision (CV) ≤ 20%, the pre‐specified acceptability criterion for functional sensitivity. Five clinical samples with initial [TA] concentrations of 1.3, 1.5, 2.1, 1.7, and 2.1 μg/mL were serially diluted (1:2, 1:4, 1:8) in normal saline. For each dilution, 10 replicates were analyzed. Serial dilution was continued until the observed CV exceeded 20%. The LLoQ was determined by interpolation of the concentration corresponding to a CV of 20%.

### 2.6 Statistical Analysis

Mean values, standard deviation, and coefficient of variability (CV%) were calculated for each dataset as applicable. Percent recovery was computed for dilution and freeze–thaw experiments to evaluate assay robustness. Linearity under dilution was assessed using linear regression of expected versus measured concentrations across serial dilutions, with goodness‐of‐fit quantified by the coefficient of determination (*R* ^2^).

To evaluate the correlation between the ELISA assay for [HMWA] and the turbidimetric immunoassay for [TA], Spearman's rank test was used. The HMWA:TA ratio was calculated to explore proportional relationships. Agreement between assays was further assessed using Passing‐Bablok regression, which quantified systematic bias (intercept) and proportional bias (slope). This regression model was selected for its robustness to outliers and lack of distributional assumptions. The resulting linear equation was used to derive a conversion factor between assays.

The lower limit of quantification (LLoQ) was interpolated using a mixed effects regression model to account for repeated measures and assay variability, consistent with approaches recommended for bioanalytical method validation and longitudinal modeling [20]. No observations were identified or excluded as outliers during analysis. Missing or indeterminate data were handled via listwise deletion, a standard approach used by the STATA statistical package.

All analyses were performed using Stata 18MP (StataCorp, College Station, TX) and Prism 10 (GraphPad Software, Boston, MA). Statistical tests were two‐sided, with a significance threshold of *p* < 0.05. Confidence intervals were reported for all key estimates where applicable.

## 3 Results

For the intra‐assay precision assessment, the first experiment analyzing 20 same‐day replicates of two samples, the sample means were 5.5 μg/mL (CV 2.8%) and 9.0 μg/mL (CV 1.8%) (Table 1). In the second experiment, intra‐assay precision of 10 replicates of 5 concentrations, testing generated an overall mean intra‐assay CV of 2.5% (Table 2).

**TABLE 1:** Intra‐ and inter‐assay variability in equine serum total adiponectin concentrations. Values are shown for 20 replicates of 2 serum samples bracketing the clinical decision point of 7.9 μg/mL analyzed on the same day (left‐hand columns), and 2 different samples analyzed over 20 consecutive days (right‐hand columns). The mean values (mean), standard deviation (SD), and coefficients of variation (CV%) are noted in bold.

Replicate #	Intra‐assay variability (within‐day)		Inter‐assay variability (between‐days)	
	1	2	3	4
1	5.7	9.1	5.9	11.2
2	5.7	9.2	5.5	10.2
3	5.7	9.1	5.6	10.4
4	5.7	8.8	5.8	11.1
5	5.6	8.9	6.0	10.9
6	5.6	9.2	6.0	11.5
7	5.5	8.9	5.8	10.7
8	5.7	8.9	5.9	10.8
9	5.4	9.0	5.6	10.6
10	5.4	9.0	6.1	11.6
11	5.4	9.0	5.8	10.6
12	5.4	8.8	5.9	10.7
13	5.4	8.8	5.8	10.7
14	5.3	8.9	5.5	10.2
15	5.3	8.9	5.9	10.8
16	5.3	9.3	5.8	10.7
17	5.4	9.0	5.9	10.5
18	5.3	9.2	5.8	10.6
19	5.6	9.3	6.2	10.8
20	5.6	9.1	6.0	11.0
Mean	**5.5**	**9.0**	**5.8**	**10.8**
SD	**0.156**	**0.161**	**0.185**	**0.366**
CV%	**2.8%**	**1.8%**	**3.2%**	**3.4%**

**TABLE 2:** Intra‐assay variability in equine serum total adiponectin concentrations [TA]. Values are shown for 10 replicates of 5 serum samples, ranging from very low (< 4 μg/mL), mid (8–10 μg/mL), and very high (> 12 μg/mL) [total adiponectin]. The mean values, standard deviation (SD), and coefficients of variation (CV%) are noted in bold.

[TA]	Very low < 4	Low, 4–8	Mid, 8–10	High, 10–12	Very high > 12
1	1.1	4.3	9.0	9.9	18.1
2	1.0	4.2	8.7	10.1	18.3
3	1.0	4.3	8.8	9.9	18.7
4	1.1	4.2	8.7	9.9	18.1
5	1.0	4.2	8.5	10	18.6
6	1.0	4.3	8.6	9.8	18.8
7	1.0	4.4	8.6	10	19.3
8	1.0	4.3	8.4	9.8	18.8
9	1.0	4.3	8.5	9.9	18.4
10	0.9	4.2	8.7	10.1	18.7
Mean	**1.0**	**4.3**	**8.7**	**9.9**	**18.6**
SD	**0.057**	**0.066**	**0.172**	**0.108**	**0.368**
CV%	**5.6%**	**1.6%**	**2.0%**	**1.1%**	**2.0%**

For the inter‐assay (across‐days) precision testing of 2 samples measured 20 times over 20 consecutive days, the [TA] mean values were 5.8 μg/mL (CV 3.2%) and 10.8 μg/mL (CV 3.4%) (Table 1). For inter‐assay testing across reagent lots (across‐lots), the mean CV was 11.7% with a range of 9.0%–17.9% (Table 3).

**TABLE 3:** Inter‐assay variability (cross‐lots) in equine serum total adiponectin concentrations. Values are shown for 4 replicates of 5 serum samples ranging from 2.4–18.6 μg/mL [total adiponectin], using different reagent lots at each time point. The mean values (mean), standard deviation (SD), and coefficients of variation (CV%) are noted in bold.

Replicate #	1	2	3	4	Mean	SD	CV%
Sample 1	3.0	2.2	2.0	2.5	**2.4**	**0.435**	**17.9%**
Sample 2	4.3	3.5	3.6	4.1	**3.9**	**0.375**	**9.7%**
Sample 3	8.7	11.3	10.7	11.2	**10.5**	**1.237**	**11.8%**
Sample 4	9.9	12.4	11	12.1	**11.4**	**1.122**	**9.9%**
Sample 5	18.6	21.8	19	22.1	**20.4**	**1.837**	**9.0%**

Table 4 shows the expected (by calculation) and measured recovery of [TA] in samples diluted with normal saline; mean recovery was 133.7%, with a linearity under dilution of *R* ^2^ = 0.99 (*p* < 0.0001; Figure 1). The CV for 5 freeze–thaw cycles was between 1.0% and 3.0%, with a median change in concentration of −13% after 1 freeze–thaw cycle, and a mean change in concentration of +1.3% in the cycles performed after the initial freeze–thaw (Table 5). Serial dilutions were performed at 1:2, 1:4, and 1:8, by which point analysis of 4 of the 5 samples revealed a CV > 20%. With undiluted samples, 10 replicates of 5 low concentration samples yielded a median CV of 3.4% (median SD 0.070); the median CV and SD at 1:2, 1:4, and 1:8 dilutions were 7.3% (0.084), 11.3% (0.070), and 29.3% (0.087). Interpolation of these values via mixed effects regression yielded an LLoQ of 1.3 μg/mL (95% confidence interval (CI) 0.57–2.04, *p* < 0.0001).

**TABLE 4:** Recovery upon dilution of total adiponectin was assessed by diluting 4 equine serum samples of low (4.8 μg/mL), mid (8–10 μg/mL), high (10–12 μg/mL), and very high (> 12 μg/mL) [total adiponectin] at baseline (undiluted) and diluted with normal saline at ratios from 2:1 to 1:6. The expected value (as calculated) was compared to the obtained result, and the percent recovery was calculated. The mean % recovery for each concentration is noted in bold.

	Low 4–8 μg/mL			Mid 8–10 μg/mL			High 10–12 μg/mL			Very high > 12 μg/mL		
	Expected	Result	% recovery	Expected	Result	% recovery	Expected	Result	% recovery	Expected	Result	% recovery
Neat	4.3			8.7			9.9			18.6		
2:1	2.9	3.3	115.1%	5.8	6.5	112.1%	6.6	7.4	112.1%	12.4	13.2	106.5%
1:1	2.2	2.7	125.6%	4.4	5.3	121.8%	5.0	6	121.2%	9.3	10.3	110.8%
1:2	1.4	2	139.5%	2.9	3.9	134.5%	3.3	4.5	136.4%	6.2	7.5	121.0%
1:4	0.9	1.1	127.9%	1.7	2.6	149.4%	2.0	3.1	156.6%	3.7	4.9	131.7%
1:6	0.6	0.9	146.5%	1.2	2.3	185.1%	1.4	2.5	176.8%	2.7	3.8	143.0%
Mean recovery %			130.9%			140.6%			140.6%			122.6%

**FIGURE 1:**
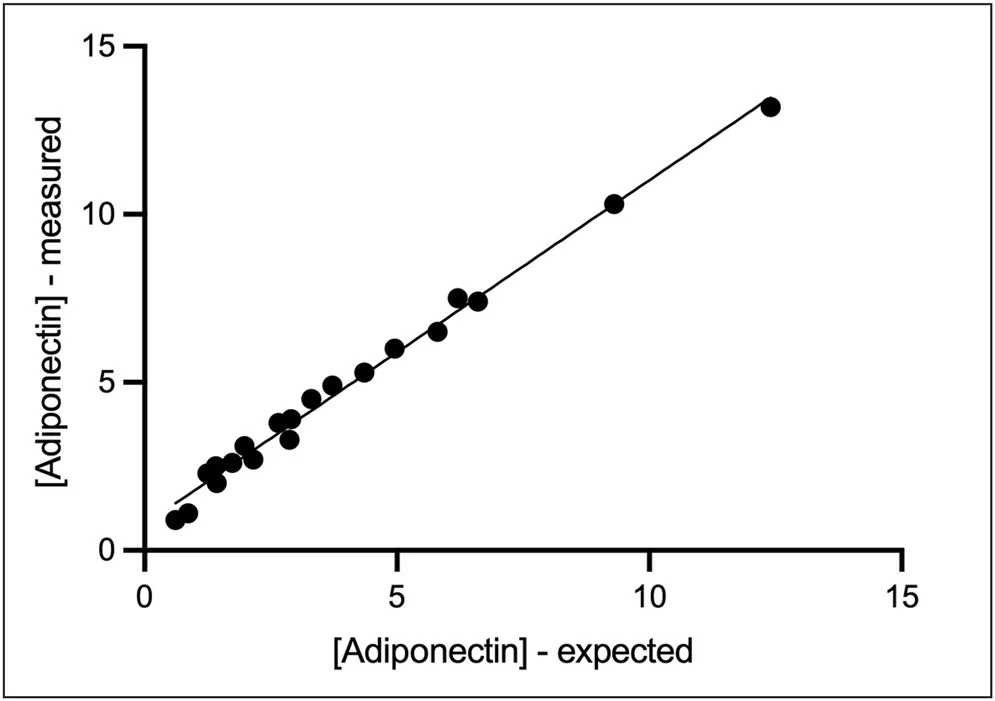
Linearity of expected versus measured total adiponectin concentrations (μg/mL) of 3 equine serum samples, serially diluted with normal saline. Goodness‐of‐fit *R* ^2^ = 0.991 (*p* < 0.0001).

**TABLE 5:** To assess freeze–thaw stability, 3 equine serum samples with very low (< 4 μg/mL), mid (8–10 μg/mL), and very high (> 12 μg/mL) [total adiponectin] were aliquoted and frozen at −80°C. Over 5 days, the aliquots underwent 5 freeze–thaw cycles and were re‐analyzed. The mean values (mean), standard deviation (SD), and coefficients of variation (CV%) are noted in bold.

Freeze/thaw #	Low 4–8	% change	Mid 8–10	% change	Very high > 12	% change
0 (before freeze)	3.4		8.9		13.6	
1	2.9	−14.7%	7.7	−13.5%	12.0	−11.8%
2	2.7	−20.6%	7.9	−11.2%	12.1	−11.0%
3	2.8	−17.6%	8	−10.1%	12.0	−11.8%
4	2.9	−14.7%	8.3	−6.7%	12.1	−11.0%
5	2.8	−17.6%	8.2	−7.9%	12.3	−9.6%
Mean	**2.8**	**−17.1%**	**8.0**	**−9.9%**	**12.1**	**−11.0%**
SD	**0.084**		**0.239**		**0.122**	
CV%	**3.0%**		**3.0%**		**1.0%**	

The mean [TA] of 207 equine serum samples was 8.87 μg/mL (SD 5.12; range 1.20–29.49 μg/mL); for the same samples, the mean [HMWA] was 6.39 μg/mL (SD 7.14; range 0–37.42 μg/mL). Spearman's rank correlation coefficient between [HMWA] and [TA] was *ρ* = 0.90 (95% CI 0.87–0.92; *p* < 0.0001). Passing–Bablok regression of the full dataset demonstrated both a systematic bias of −4.61 μg/mL and a proportional bias of 1.24, indicating that [HMWA] tended to be higher than [TA], especially at higher concentrations ([HMWA] = −4.61 + 1.24*[TA]). After excluding samples with [HMWA] > 10 μg/mL, the regression revealed a systematic bias of −2.83 μg/mL and a proportional bias of 0.88, indicating lower values of [HMWA] than [TA] at lower concentrations ([HMWA < 10] = −2.83 + 0.88*[TA]; Figure 2). The mean ratio of HMWA:TA was 0.60 (SD 0.401) for 191 samples, excluding 16 samples where HMWA = 0 μg/mL. However, 37 of the 207 (18%) [HMWA] values were higher than the [TA] value, leading to a biologically spurious ratio > 1. Of this subset of samples, only 1/37 had a [HMWA] of < 7.9 μg/mL, and the mean [HMWA] of these samples was 17.7 μg/mL.

**FIGURE 2:**
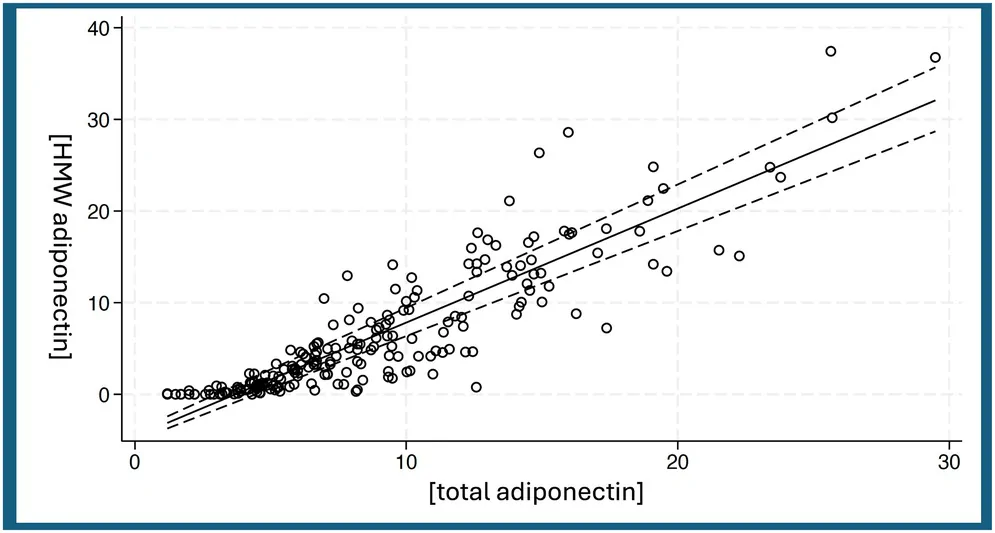
Passing‐Bablok regression of [total adiponectin] of equine serum (*n* = 207, range 1.2–29.49 μg/mL) measured with Immunodiagnostics Adiponectin Turbidimetric Immunoassay, plotted against high molecular weight (HMW) adiponectin quantified using the MilliporeSigma HMW Adiponectin ELISA (*n* = 207, range 0–37.42 μg/mL). The solid line represents the line of best fit (dashed lines, 95% confidence interval). In this figure, the x‐intercept indicates systematic (constant) bias, and the slope, proportional bias.

## 4 Discussion

This study demonstrates that the Immunodiagnostics Adiponectin Turbidimetric Immunoassay Kit exhibits adequate precision and reliability, with good intra‐ and inter‐assay repeatability and reproducibility, and CV less than 5% for both within‐day and between‐day replicates. This is similar to intra‐assay and inter‐assay results of the human Randox total adiponectin IT assay when validated for equine samples, with CV of 1.02% and 1.87% respectively [21]. Inter‐assay variability using different reagent lots and performed on frozen samples over a 10‐month period showed a greater variance, with samples bracketing the critical clinical decision point of 7.9 μg/mL exhibiting CV of approximately 10%. However, CV was considerably higher for a lower concentration sample close to the low‐end 2.5 μg/mL cutoff, suggesting that values around this range may be less precise. These findings together suggest that the variation in reagent lot or other analytical factors secondary to long inter‐assay periods create more imprecision that the freeze–thaw effect itself; this should be considered when making serial measurement on a patient that span over reagent lots, as would happen if re‐testing is performed semiannually or annually. A limitation of this study is that specific method‐validation guidelines (e.g., CLSI EP05‐A3, EP17‐A2) were not used to define acceptance criteria for each parameter.

The value of LLoQ we obtained falls below the lowest diagnostic cut point and supports this assay as having a functional range acceptable for the measurement of adiponectin in equine serum samples. Most references indicate that values below 4 μg/mL indicate markedly increased laminitis risk; high precision below the LLoQ of this assay would not provide additional clinical benefit. Linearity when assaying serially diluted samples was excellent, with moderate increases in recovery that were grossly similar between low and very high concentration sera, likely due to matrix interference. Sources of matrix interference in horses may include heterophilic antibodies, labile endogenous inhibitors, and poor matrix matching between equine serum and the saline diluent. This indicates that there may not be a significant hook effect, an artifactual undermeasuring of samples with high antigen load [22], at very high physiologic concentrations.

Adiponectin concentrations are generally considered to be stable for at least 36 h of refrigeration [23], and after long‐term freezing (even for decades) [21], and after multiple freeze–thaw cycles [24]. In our samples, the TA concentration of samples dropped by approximately 13% after a single freeze–thaw cycle. The effect did not appear to be additive with additional freeze–thaw cycles up to 5, a finding which was consistent across all 3 concentrations tested. This suggests that banked samples stored at −80°C are generally suitable for analysis, although samples with concentrations close to a cutoff value may be misclassified in thawed sera. However, in the authors' laboratory, a series of ranges is used to stratify risk of laminitis as significant values of hypoadiponectinemia may differ between breeds (light horses versus warmbloods versus ponies), with values exceeding 10 μg/mL associated with low risk of laminitis in all breeds, 8–10 μg/mL as equivocal, 6–8 μg/mL indicating mild metabolic dysfunction, 4–6 indicating moderate metabolic dysfunction with increased risk of laminitis, and < 4 μg/mL evidencing severe metabolic dysfunction with high risk of laminitis.

Overall, results from the TA assay showed adequate correlation (*ρ* = 0.90) with those of the HMWA assay, as has been noted in previous studies in humans, with reported correlation coefficients of *ρ* = 0.9725 and *ρ* = 0.9826, suggesting that these fractions may be regulated synchronously in humans. However, in this study the ratios of HMWA:TA in individual samples often differed greatly; in almost a fifth of samples, [HMWA] exceeded [TA], a result which is evidently biologically spurious. However, in all but one sample, this finding was limited to sera with high HMWA concentrations. In samples with HMWA:TA ratio > 1.0, the mean value of [HMWA] was 17.7 μg/mL, which suggests that either the HMWA assay is inaccurate at higher ranges or that the TA assay provides falsely low values in high concentration samples.

Approximately 18% of samples in this study had calculated [HMWA] values that exceeded [TA], which is clearly biologically impossible, and thus reflects analytical artifact. Potential contributors include antibody specificity differences between the total and HMW assays, differences in calibrator material, sample pre‐treatment steps required for HMWA release that may alter recovery differently than the native TA assay, and/or high‐end underestimation of TA due to non‐linearity or hook effects. The absence of an internationally recognized standard for either TA or HMWA means that calibration is assay‐specific, and that absolute values and derived ratios are not directly comparable across platforms. These findings emphasize that HMWA:TA ratios should not be interpreted as absolute physiologic values across platforms, but rather as platform‐specific derived indices that may be sensitive to analytical bias at both ends of the numerator and denominator.

The HMWA:TA ratio of 0.60 noted in the samples from the current study is similar to that reported in humans, 0.76 ± 0.14 in healthy women and 0.62 ± 0.12 in healthy men [25]. It is posited that the mechanism for higher HMWA:TA ratios in women versus men is that testosterone suppresses the secretion of HMWA [26]. This may explain a component of the well‐described increased risk for insulin resistance in men compared to women [27]. Investigating the sexual dimorphism of the HMWA:TA ratio and its potential as a marker of the biological activity of adiponectin in equids may be worth future research efforts.

Overall, these experiments indicate that the Immunodiagnostics Adiponectin Turbidimetric Immunoassay Kit is suitable for clinical use for the determination of [TA] in equine serum samples. The assay is rapid, inexpensive, and readily automated, and provides important data for clinicians to assess the risk of laminitis and monitor therapeutic interventions of equine metabolic disease. Caution is warranted when assessing frozen/thawed samples, and long‐term serial monitoring where samples are likely to be tested using different reagent lots in samples that are close to critical cut‐off values. Additional studies, both in breed differences and biologic variation of [TA] in equids, would be beneficial both in interpreting results across a variety of breed phenotypes as well as constructing reference change values to allow assessment of the significance of differences between serial measurements.

## Funding

This work was supported by the Laminitis Research Fund, University of Pennsylvania.

## Conflicts of Interest

The authors declare no conflicts of interest.

## Data Availability

The data that support the findings of this study are available from the corresponding author upon reasonable request.
